# People with type 2 diabetes and screen-detected cognitive impairment use acute health care services more often: observations from the COG-ID study

**DOI:** 10.1186/s13098-019-0416-z

**Published:** 2019-02-22

**Authors:** Jolien Janssen, Paula S. Koekkoek, Geert Jan Biessels, L. Jaap Kappelle, Guy E. H. M. Rutten, the Cog-ID study group, Minke Kooistra, Esther van den Berg, J. Matthijs Biesbroek, Onno Groeneveld

**Affiliations:** 1 Julius Centre for Health Sciences and Primary Care, University Medical Centre Utrecht, Utrecht, The Netherlands; 2 Department of Neurology, Brain Centre Rudolf Magnus, University Medical Centre Utrecht, Utrecht, The Netherlands

**Keywords:** Cognitive impairment, Diabetes, Screening, General practice, Acute health care services, Falls

## Abstract

**Background:**

Patients with type 2 diabetes have an increased risk of cognitive impairment which can lead to impaired diabetes self-management and an increased risk of diabetes-related complications. Routine screening for cognitive impairment in elderly patients with type 2 diabetes is therefore increasingly advocated. The aim of this study is to investigate whether people with type 2 diabetes and screen-detected cognitive impairment use acute health care services more often than patients not suspected of cognitive impairment.

**Methods:**

People with type 2 diabetes ≥ 70 years were screened for cognitive impairment in primary care. Diagnoses in screen positives were established at a memory clinic. Information about acute health care use was collected for 2 years prior to and 2 years after screening and compared to screen negatives.

**Results:**

154 participants (38% female, mean age 76.7 ± 5.2 years, diabetes duration 8.7 ± 8.2 years) were included, 37 patients with cognitive impairment, 117 screen negatives. A higher percentage of participants with cognitive impairment compared to screen negative patients used acute health care services; this difference was significant for general practitioner’s out of hours services (56% versus 34% used this service over 4 years, p = 0.02). The mean number of acute health care visits was also higher in those with cognitive impairment than in screen negatives (2.2 ± 2.8 versus 1.4 ± 2.2 visits in 4 years, p < 0.05; 1.4 ± 2.2 versus 0.7 ± 1.5 visits in 2 years after screening, p = 0.03). Factors that could have played a role in this increased risk of acute health care services use were a low educational level, the presence of depressive symptoms (CES-D score ≥ 16), self-reported problems in self-care and self-reported problems in usual activities.

**Conclusions:**

People with type 2 diabetes and screen-detected cognitive impairment use acute health care services more often.

**Electronic supplementary material:**

The online version of this article (10.1186/s13098-019-0416-z) contains supplementary material, which is available to authorized users.

## Background

Patients with type 2 diabetes have an increased risk of cognitive impairment and dementia [1, 2]. Cognitive impairment, already in its early stages, can lead to impaired diabetes self-management [3, 4]. Patients with diabetes and cognitive impairment have increased risks of hypoglycemic events, cardiovascular events and even death compared to those without cognitive impairment [5–7]. In addition, cognitive impairment in diabetes is associated with a reduced health status and more depressive symptoms [8]. Therefore, recent guidelines recommend individualized diabetes treatment for patients with cognitive impairment [9].

Since cognitive impairment often remains unrecognized [10–12], routine screening for cognitive impairment in elderly patients with type 2 diabetes is increasingly advocated [9]. The argument is that routine screening may identify patients with cognitive impairment who might then benefit from a personalized intervention. It is however unknown how often people with type 2 diabetes and cognitive impairment identified through screening (screen-detected cognitive impairment) experience acute health problems (e.g. problems that require the use of acute health care services or falls) and if this is indeed more often than patients without cognitive impairment.

The Cognitive Impairment in Diabetes (Cog-ID) study aimed to establish a primary care based screening strategy to detect cognitive impairment [13]. The study showed that self-administered cognitive screening tests can be used for this purpose and that the Self-Administered Gerocognitive Examination (SAGE) had the best diagnostic accuracy (negative predictive value of 85%; positive predictive value of 40%) with a memory clinic established diagnosis as reference standard. Because health outcomes were recorded for the 2 years prior to and after screening, the Cog-ID study is ideally suited to investigate whether people with type 2 diabetes and screen-detected cognitive impairment use acute health care services more often and if they report more falls than people without cognitive impairment.

## Methods

### Design

The design of the Cog-ID study has been described in detail elsewhere [13]. In brief, people ≥ 70 years with type 2 diabetes were invited to participate by their general practitioner (GP) in the period August 2012 to September 2014. People with a previous diagnosis of dementia, a previous memory clinic evaluation or the inability to write or read Dutch were excluded. Written informed consent was obtained from all participants.

**Fig. 1:**
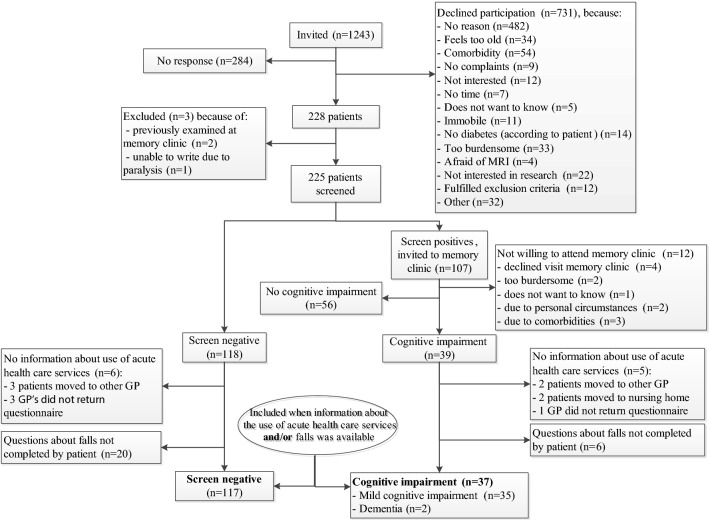
Patient flow

Participants were first visited at home by a research physician who screened for cognitive impairment with two self-administered cognitive tests (the SAGE and the ‘Test Your Memory’ (TYM)), the Mini-Mental state examination (MMSE) and a structured interview. People who were not suspected of cognitive impairment based on this screening visit are referred to as ‘screen negatives’ and those suspected of cognitive impairment as ‘screen positives’. Screen positives received a standardized memory clinic evaluation as reference standard. Screen positives who fulfilled criteria for mild cognitive impairment (MCI) or dementia were subsequently diagnosed with cognitive impairment. The current study includes the screen positive patients diagnosed with cognitive impairment and all screen negative patients (Fig. 1).

The GPs of patients diagnosed with cognitive impairment at the memory clinic received information about the diagnosis, that was accompanied by a letter with a not binding advice on how to tailor patient’s diabetes care in light of the cognitive problems (Additional file 1).

### Use of acute health care services

Short questionnaires were sent to all general practices to collect information about the use of acute health care services, defined as any of the following: unplanned hospitalizations, emergency room visits and visits to GP out of hours services (between 5.00 p.m. and 8.00 a.m.). Consecutive acute health care visits within 48 h for the same health problem were counted as one acute health care visit, e.g. when patients consulted the emergency room and were hospitalized 1 or 2 days afterwards. Calls to the GP out of hours services were not included. Hospitalizations were categorized as ‘unplanned’ (= acute) and ‘other’ (= not acute), as shown in Additional file 2. Unplanned hospitalizations were defined as ‘an unexpected admission for the management of a disease or treatment-related event that cannot be controlled in the outpatient setting’. Patients who died within 24 months after screening were not excluded for the analysis, their use of acute health care services was registered until the day of their death.

### Falls

Twenty-four months after the home screening visit participants received a follow-up questionnaire with the following questions, namely (1) ‘Did you fall in the past year?’ (yes or no) and (2) ‘If yes, how many times did you fall in the past year?’. We chose to ask patients only about falls in the past year and not about falls in the past 2 years to minimize the risk of memory bias. Falls in the years prior to screening were not registered.

### General practitioner questionnaires

To evaluate if and how GP’s changed their patient’s treatment after a diagnosis of cognitive impairment, we sent a questionnaire to the GPs with the following questions: (1) ’Did the result of the memory clinic came as a surprise for you?’ (yes/no); (2) ‘Did you change your patient’s diabetes treatment as a result of the diagnosis of cognitive impairment?’ (yes/no and open field) and (3) ‘Did the results of the screening and the possible diagnosis of cognitive impairment have implications for the patient’s treatment, that are not related to their diabetes?’ (yes/no and open field).

### Other measures

During the (screening) visit at home by the research physician, participants also completed questionnaires about depressive symptoms and health related quality of life (HRQOL). Depressive symptoms were assessed with the Centre for Epidemiologic Studies Depression Scale (CES-D). A score ≥ 16 is generally accepted as the cut-off score for the presence of depression. The European Quality of Life-5 Dimensions (EQ-5D) covers five dimensions of HRQOL: mobility, self-care, daily activities, pain/discomfort and anxiety/depression.

Information about age, sex and educational level was gathered during the home screening visit. Information about participant’s medication use, medical history, diabetes duration, BMI, MDRD and HbA1c was collected by the researchers from the participant’s GP electronic medical record. HbA1c and MDRD values closest to the screening visit were taken, this could be up to 6 months prior or after the visit.

### Statistical analysis

Our primary aim was to describe the differences between people with and without screen-detected cognitive impairment with regard to the use of acute health care services and not to model determinants of acute health care use. The proportion of patients with at least one time use of an acute health care service was compared between those with screen-detected cognitive impairment and screen negative patients with a Chi square test. The mean number of acute health care visits was compared between the groups with a Mann–Whitney-U-test. The same tests were used to investigate fall accidents.

In addition, the proportion of patients with at least one time use of an acute health care service was compared between the years prior to and the years after screening using a Mc Nemar test, for each of the groups separately. The mean number of acute health care visits was compared between the years prior to and the years after screening with a Wilcoxon Signed-Rank-test, for each of the groups separately. The Mann–Whitney-U-test was used to test whether this increase or decrease in mean number of acute health care visits differed between the groups.

To explore whether other factors than cognitive impairment could explain between group differences, we looked whether the use of acute health care services differed between groups that were stratified based on baseline characteristics with an unequal distribution between the groups.

A *p* value ≤ 0.05 was considered significant. All statistical analyses were performed using IBM SPSS statistics 21

## Results

### Study population

Of the 1243 patients eligible for the COG-ID study, 731 declined participation and 284 did not respond to the invitation (Fig. 1). Of the 225 patients who participated and were screened for cognitive impairment, 118 were screen negative. Of the 107 patients who were screen positive, 39 were diagnosed with cognitive impairment at the memory clinic. Of the remaining screen positives, 12 were not willing to attend the memory clinic and 56 had no cognitive impairment compatible with MCI or dementia criteria; these patients were not included in the current analysis. Three patients (two with cognitive impairment, one screen negative patient) with missing information about both the use of acute heath care services and about falls were not included in the current analyses (Fig. 1). The remaining 37 patients with cognitive impairment and 117 screen negative patients were included in this study, resulting in a study population of 154 individuals. Their baseline characteristics are summarized in Table 1.

**Table 1:** Characteristics of participants at time of screening

	Total study population (n = 154)	Screen-detected cognitive impairment (n = 37)	Screen negatives (n = 117)
Age (years)	76.7 ± 5.2	77.8 ± 5.6	76.4 ± 5.0
Female sex	58 (38%)	15 (41%)	43 (37%)
Living alone	57 (37%)	10 (27%)	47 (40%)
Educational level^a^	5 (4–6)	4 (2–5)*	5 (5–6)*
Low educational level (Verhage scale 1–4)	46 (30%)	22 (60%)*	24 (20%)*
Diabetes duration (years)	8.7 ± 8.2	10.6 ± 8.1	8.1 ± 8.1
HbA1c (mmol/mol)	52.2 ± 9.7	53.8 ± 9.8	51.7 ± 9.6
HbA1c (%)	6.9 ± 0.9	7.1 ± 0.9	6.9 ± 0.9
Use of Metformin, yes	104 (78%)	22 (76%)	82 (80%)
Use of insulin, yes	30 (20%)	9 (24%)	21 (18%)
Use of Sulfonylurea, yes	45 (29%)	9 (24%)	36 (31%)
Use of lipid lowering drugs, yes	122 (80%)	29 (78%)	93 (81%)
Diabetic neuropathy, yes	15 (10%)	5 (14%)	10 (9%)
Diabetic retinopathy, yes	11 (7%)	4 (11%)	7 (6%)
MDRD	67.9 ± 19.2	64.9 ± 20.7	71.9 ± 18.5
BMI (kg/m^2^)	28.6 ± 4.4	29.2 ± 4.8	28.4 ± 4.3
Systolic blood pressure (mm Hg)	139.8 ± 17.4	140.4 ± 13.3	139.6 ± 18.6
Diastolic blood pressure (mm Hg)	75.4 ± 11.4	76.0 ± 12.1	75.3 ± 11.2
MMSE	28.4 ± 2.0	26.4 ± 3.0*	29.0 ± 1.1*
TYM score	42.4 ± 6.4	35.4 ± 8.8*	44.5 ± 2.6*
SAGE score	17.1 ± 4.1	11.5 ± 4.4*	18.6 ± 2.2*
Equation 5D mobility, any problems (%)	83 (55%)	24 (65%)	59 (51%)
Equation 5D self care, any problems (%)	17 (11%)	8 (22%)*	9 (8%)*
Equation 5D usual activities, any problems (%)	49 (32%)	22 (59%)*	27 (23%)*
CES-D ≥ 16	27 (18%)	13 (36%)*	14 (12%)*

Data are presented as means (± standard deviation), median (interquartile range), or number and proportion in  %

*BMI* body mass index, *CES-D* Centre for Epidemiologic Studies Depression Scale, *Equation* *5D* EuroQol Five-Dimension Scale, *MDRD* modification of diet in renal disease, *MMSE* Mini-Mental state examination, *TYM* Test Your Memory, *SAGE* Self-Administered Gerocognitive Examination

* p ≤ 0.05 for comparison between the groups (Chi square test/t-test)

^a^Educational level is classified by the Dutch Verhage scale [24]; a seven point rating scale ranging from 1 (which equals a level of less than 6 years of elementary school) to 7 (equals a finished training at a university or technical college)

Mean age was 76.7 ± 5.2 years, 58 (38%) were female and 57 (37%) were living alone. The mean duration of diabetes was 8.7 ± 8.2 years, mean HbA1c level 52.2 ± 9.7 mmol/l (6.9 ± 0.9%) and 30 (20%) of the patients used insulin. A higher percentage of people with screen-detected cognitive impairment had a low educational level, depressive symptoms, problems with self-care and problems with usual activities. In addition, this group had also lower MMSE, TYME and SAGE scores compared to the screen-negative participants (Table 1). Two (5%) patients with cognitive impairment and six (5%) of the screen negative patients died within 2 years after screening.

### Use of acute health care services

**Fig. 2:**
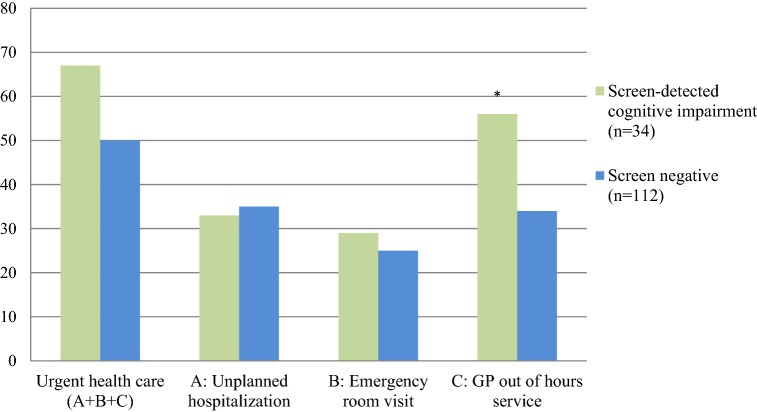
Percentage patients that used the acute health care service at least once in 4 years.* p ≤ 0.05 for the difference in proportion of patients with at least one time use of an acute health care service. *GP* General practitioner

As shown in Fig. 2, more participants with cognitive impairment than screen negative patients used acute health care services, this difference between the groups was only significant for general practitioners out of hours services (56% versus 34% used this service over 4 years, p = 0.02).

The mean number of all acute health care visits and unplanned hospital admissions was significantly higher in those with cognitive impairment than in screen negative patients, both in the total 4 year period (2.2 ± 2.8 versus 1.4 ± 2.2, p < 0.05) and in the 2 years after screening (1.4 ± 2.2 versus 0.8 ± 1.4, p = 0.03), as depicted in Table 2. Again, this was most evident for visits to GP out of hours services. The mean number of GP out of hours visits was significantly higher in patients with cognitive impairment than that in screen negative patients (1.4 ± 1.8 versus 0.7 ± 1.3 visits over the total 4 years, p = 0.01; 0.8 ± 1.4 versus 0.3 ± 0.8 over the 2 years after screening, p = 0.03).

**Table 2:** Mean number of acute health care visits

	Screen-detected cognitive impairment (n = 34)			Screen negative (n = 112)		
	4 year period	2 years prior	2 years after	4 year period	2 years prior	2 years after
Acute health care services (A + B+C)	2.2 ± 2.8*	0.8 ± 1.2	1.4 ± 2.2*	1.4 ± 2.2*	0.7 ± 1.2	0.7 ± 1.5*
A: Unplanned hospitalization	0.6 ± 1.2	0.2 ± 0.6	0.5 ± 1.0	0.6 ± 1.1	0.3 ± 0.6	0.3 ± 0.8
B: Emergency room visit	0.6 ± 1.1	0.2 ± 0.7	0.4 ± 0.7	0.4 ± 0.8	0.2 ± 0.6	0.2 ± 0.5
C: GP out of hours service	1.4 ± 1.8*	0.6 ± 0.9	0.8 ± 1.4*	0.7 ± 1.3*	0.4 ± 0.8	0.3 ± 0.8*

* p ≤ 0.05 for difference in mean number of acute health care visits between screen negatives and those with screen-detected cognitive impairment

**Fig. 3:**
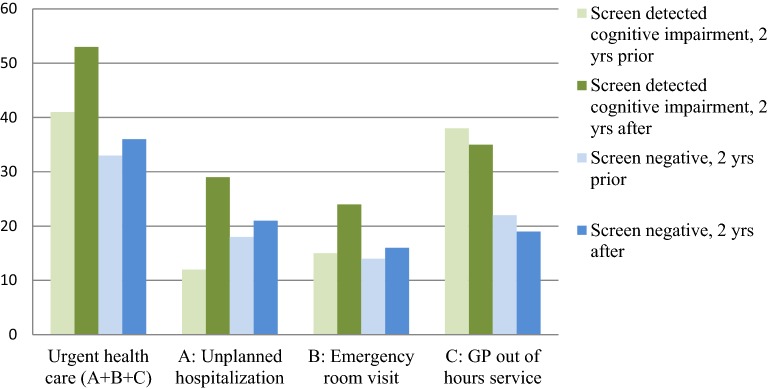
Percentage of patients that used the acute health care service at least once in the 2 years prior and in the 2 years after screening. *GP* General practitioner

Comparing the years after to the years prior to screening for each of the groups separately, there was no significant increase or decrease in the use of acute health care services. These changes (increase or decrease) in the use of acute health care services did also not differ significantly between the two groups (Table 2 and Fig. 3). Table 3 shows that, people with or without cognitive impairment and a relatively low educational level, or with self-reported problems in self-care, or with self-reported problems in usual activities or with depressive symptoms all tend to use acute health care services more often.

**Table 3:** Living situation, educational level, EQ5D and CES-D and the use of acute health care services in the total study population

	Living situation		Educational level		EQ-5D self-care		EQ-5D usual activities		CES-D	
	Alone (n = 54)	With others (n = 92)	1–4^a^ (n = 43)	5–7^a^ (n = 103)	Any problem (n = 13)	No problem (n = 132)	Any problem (n = 45)	No problem (n = 101)	≥ 16 (n = 24)	<16 (n = 121)
N(%) people that used GP out of hour services at least once in 4 years	25 (46%)	32 (35%)	21 (49%)	36 (35%)	8 (62%)	48 (36%)	24 (53%)	33 (33%)	13 (54%)	43 (36%)
Mean (± SD) number of visits to GP out of hour services in 4 years	0.8 ± 1.3	0.8 ± 1.6	1.2 ± 2.2	0.5 ± 0.9	1.5 ± 1.9	0.8 ± 1.4	1.4 ± 2.1	0.6 ± 1.0	1.5 ± 2.0	0.7 ± 1.3
Mean number (± SD) of acute health care visits in 4 years	1.6 ± 1.8	1.5 ± 2.6	2.3 ± 3.4	1.2 ± 1.7	2.5 ± 2.3	1.5 ± 2.4	2.5 ± 3.0	1.1 ± 1.9	2.9 ± 3.4	1.3 ± 2.1

Data are presented as means (± standard deviation) or number and proportion in  %. *GP* general practitioner, *CES-D* Centre for Epidemiologic Studies Depression Scale, *EQ-5D* European Quality of Life-5 Dimensions

^a^Educational level is classified by the Dutch Verhage scale [24]; a seven point rating scale ranging from 1 (which equals a level of less than 6 years of elementary school) to 7 (equals a finished training at a university or technical college)

### Falls

Twelve patients with cognitive impairment (36%) and 24 (25%) screen negative people reported at least one fall accident in the 12 to 24 months after screening (p = 0.186). The mean number of falls in that period did not differ between both groups (1.9 ± 4.6 versus 0.7 ± 1.7, p = 0.176).

### General practitioner questionnaires

In eleven (28%) of the 39 patients with screen-detected cognitive impairment their GP had not suspected the diagnosis. Only two (5%) GPs changed their patient’s diabetes treatment as a result of the diagnosis of cognitive impairment (one increased the HbA1c target, one lowered the insulin dosage). In seven (18%) cases the diagnosis had other implications (treatment discussed with patient, situation at home discussed with daughter, more care in nursing home, close monitoring of the course of cognitive function (2x) and being more alert to problems at home (2x)).

## Discussion

This study shows that patients with cognitive impairment, detected during a screening program in individuals with diabetes ≥ 70 years, more often use acute health care services than patients without cognitive impairment.

These findings are in line with previous studies that demonstrate that patients with both type 2 diabetes and cognitive impairment experience more adverse health outcomes compared to patients without cognitive impairment [5–8]. The current study shows that this increased risk is already there when patients are diagnosed with cognitive impairment by screening, even if people are diagnosed with mild cognitive impairment and not with dementian.

We explored which factors could have played a role, besides cognitive impairment. Living alone may be a reason for people not being able to visit acute health care services. Ten out of 37 (27%) participants with screen-detected cognitive impairment were living alone, compared to 47 out of 117 (40%) of the screen negatives. Table 3 shows that, in our total study population, living alone was not associated with a reduced number of visits to acute health care services and is therefore unlikely to account for the differences between the screen negatives and the screen positives. This finding is in line with a recent study among 1447 older people in the UK; those living alone had a higher probability of utilising emergency department and general practitioner services [14].

Depressive symptoms, problems with self-care and problems with usual activities were more common in those with cognitive impairment compared to the screen negatives (Table 1). Table 3 shows that both people with and without cognitive impairment but with the above mentioned problems have an increased risk of using acute health care services. This is not an unexpected finding, because these factors are interrelated with cognitive impairment. A study among 683 elderly home care recipients in Canada found significant associations between poor self-rated health, greater functional dependency and acute health care use [15]. Cognitive impairment can cause depressive symptoms and problems in self-care and usual activities, which could lead to impaired (diabetes) self- management skills and to an increased need for acute health care. Depressive symptoms, problems with self-care and problems with usual activities are therefore possible mediating factors in the association between cognitive impairment and use of acute health care services.

Low educational level is a known risk factor for cognitive impairment [16]. In addition, Table 3 shows that people with a low educational level in our study population tend to use acute health care services more often. It is therefore possible that educational level accounts for part of the differences between people with and without screen detected cognitive impairment in the utilization of acute health care services. This conclusion does not decrease the relevance of our findings, because anyway detection of cognitive impairment will identify a vulnerable patient group that may need extra attention and tailored care.

The use of acute health care services and falls are important health outcomes with a considerable impact on health expenditures, morbidity and patients’ well-being [17–19]. Therefore, our results are also relevant in light of recent American Diabetes Association (ADA) guidelines which recommend to screen elderly patients with type 2 diabetes for cognitive impairment [9]. Taken together these findings confirm the vulnerability of patients with type 2 diabetes and cognitive impairment and emphasize the importance of an individualized treatment strategy in these people.

Of note, most GPs did not adjust the diabetes treatment in patients with cognitive impairment, despite our written advice. It should be acknowledged, however, that formal guidance from organizations of health care professionals on how to manage diabetes in people with cognitive impairment was largely published after our study was performed [9]. A more active intervention is probably warranted to ensure that these guidelines are put to practice. Important points are avoiding overly intensive diabetes management and using therapies with a low risk of hypoglycaemia, as recommended by both the ADA and the Dutch College of General Practitioners [9, 20]. In clinical practise, de-intensifying glucose lowering treatment is not yet successfully implemented [21, 22].

A strength of this study is the use of a comprehensive neuropsychological assessment at the memory clinic to diagnose cognitive impairment. The response rate for the follow-up questionnaires was high; 93% of the general practitioners completed the questionnaire about acute health care visits of their patient and 83% of the participants reported about their falls after 24 months. Some limitations should also be mentioned. As shown in Fig. 1, the COG-ID participation rate was low (18%). The results of this study can therefore not be generalized to all older people with type 2 diabetes, only to those willing to participate in a screening program for cognitive impairment. In addition, we may have missed more differences between the two groups since the screening tests used in the COG-ID study do not have a sensitivity of 100%. We may assume that the group of screen negative patients included about 16% of patients with cognitive impairment [23]. However, we opted to use all screen negatives as a comparison group because a screening program for cognitive impairment in primary care will also result in false negative outcomes. Furthermore, it is possible that missing data was related to worse health status and subsequently more use of acute health services (e.g. medical records were inaccessible when the patient moved to a nursing home). This might have caused a slight underestimation of the use of acute health care in the group with most missing data, i.e. those with cognitive impairment. We could not asses the effect of the screening program and a subsequent diagnosis of cognitive impairment on acute health care use and falls, because it was not possible to compare the patients diagnosed with cognitive impairment to patients with cognitive impairment but without a diagnosis. At last, it would have been interesting to compare the number of hypoglycaemic events between the groups, however this data was not available.

## Conclusions

This study shows that elderly patients with type 2 diabetes and screen-detected cognitive impairment use acute health care services more often than patients who screened negative. These findings confirm that screening for cognitive impairment can identify a vulnerable group of patients that might benefit from more tailored care.

## Additional files

**Additional file 1.** Advice provided to the general practitioners of people diagnosed with MCI or dementia.

**Additional file 2.** Classification of unplanned and other hospitalizations.

## Abbreviations

ADA: American Diabetes Association
Cog-ID: Cognitive Impairment in Diabetes
GP: general practitioner
HbA1c: glycated haemoglobin
MCI: mild cognitive impairment
MMSE: Mini-Mental state examination
SAGE: Self-Administered Gerocognitive Examination
TYM: Test Your Memory
