# Supplementary material for: People with type 2 diabetes and screen-detected cognitive impairment use acute health care services more often: observations from the COG-ID study
Source: Diabetol Metab Syndr. 2019 Feb 22;11:21. doi: 10.1186/s13098-019-0416-z (PMC6387554; doi:10.1186/s13098-019-0416-z)
Supplement: Supplementary file 1 — Additional file 1. Advice provided to the general practitioners of people diagnosed with MCI or dementia. [file 13098_2019_416_MOESM1_ESM.docx]

**Additional file 1**

**Table –** Advice provided to the general practitioners of people diagnosed with MCI or dementia

| **Subject** | | **Advice** |
| --- | --- | --- |
| HbA1c target | Strict glycaemic control is associated with hypoglycaemic events and associated falls. This risk is even higher in people with cognitive impairment. A beneficial effect of strict glycaemic control HbA1c < 8% (64 mmol/mol) in older people and those with a long duration of diabetes is not proven. An HbA1c target around 8% (64 mmol/mol) is probably best. | |
| Prevention of hypoglycaemic events | The risk of hypoglycaemic events is higher when insulin is used, adequate use of insulin is more difficult than taking oral medication, perhaps you can replace insulin by an oral drug. | |
| Medication adherence | The use of blister packing makes it easier for people with diabetes to use multiple drugs safely, in people with cognitive impairment this might be even more important. | |
| Hyperglycaemia | If HbA1c is >10.4% (90 mmol/mol) and the patient experiences symptoms which could be due to hyperglycaemia you can explore how to support the patient with his or her treatment or to simplify the treatment. | |
| Cardiovascular risk factors | Treat other cardiovascular risk factors according to corresponding guidelines, but take into account that patient’s compliance can be affected. | |
| Reminders | Patients may forget instructions and appointments; it might help to provide notes or written instructions. | |
