# Supplementary material for: People with type 2 diabetes and screen-detected cognitive impairment use acute health care services more often: observations from the COG-ID study
Source: Diabetol Metab Syndr. 2019 Feb 22;11:21. doi: 10.1186/s13098-019-0416-z (PMC6387554; doi:10.1186/s13098-019-0416-z)
Supplement: Supplementary file 2 — Additional file 2. Classification of unplanned and other hospitalizations. [file 13098_2019_416_MOESM2_ESM.docx]

**Additional file 2**

**Table –** Unplanned and other hospitalizations

| **Unplanned hospitalizations** | **N** | **Other hospitalizations** | **N** |
| --- | --- | --- | --- |
| Asthma/COPD/pneumonia/dyspnoe | 13 | Surgery/procedure because of malignancy | 12 |
| Abdominal pain/ obstipation/diarrhoea/ileus | 9 | Cataract surgery | 10 |
| ACS | 5 | Arthrosis (joint replacement or arthrodesis) | 10 |
| Atypical thoracic pain | 4 | Insertion or replacement of ICD | 8 |
| Arrhythmia | 4 | Cholecystectomy | 3 |
| Observation/ social indication after a fall | 4 | PTA for intermittent claudication | 2 |
| Diverticulitis | 3 | Aortic valve replacement | 2 |
| Fracture hip or vertebra | 3 | Hand surgery (Dupuytren, trigger finger) | 2 |
| Urinary tract infection | 3 | CT abdomen | 2 |
| Cholecystitis | 3 | HNP surgery | 2 |
| Decompensatio cardis | 3 | Vitrectomy | 1 |
| Electrocardioversion for AF | 3 | Surgery meniscus | 1 |
| Less responsive | 2 | Maxillary surgery | 1 |
| CVA / SAB | 2 | Sinus surgery | 1 |
| Hypo/hyper kalium | 2 | Circumcision | 1 |
| Allergic reaction | 2 | Implementation ECG log | 1 |
| Gastrointestinal bleeding | 2 | Coronary Angiography | 1 |
| Infection abdominal wall | 2 | Surgery cyst dig I | 1 |
| Dysregulation DM | 1 |  |  |
| Pain hip/leg | 1 |  |  |
| Head trauma | 1 |  |  |
| Haematuria due to high INR | 1 |  |  |
| Infected kidney cyst | 1 |  |  |
| Analysis of falls, fatigue and weight loss | 1 |  |  |
| Leaking ileostomy | 1 |  |  |
| Altitude Sickness | 1 |  |  |
| Suspected arthritis | 1 |  |  |
| Infection eci | 1 |  |  |
| Surgery biceps rupture | 1 |  |  |
| PCI | 1 |  |  |
